# Celecoxib Suppresses NF-κB p65 (RelA) and TNFα Expression Signaling in Glioblastoma

**DOI:** 10.3390/jcm12206683

**Published:** 2023-10-23

**Authors:** Hina Ahsan, Shaukat Iqbal Malik, Fawad Ali Shah, Hamed A. El-Serehy, Amin Ullah, Zafar Abbas Shah

**Affiliations:** 1 Department of Bioinformatics and Biosciences, Faculty of Health and Life Sciences, Capital University of Science and Technology (CUST), Islamabad 44000, Pakistan; hina.ahsan@riphah.edu.pk; 2 Riphah Institute of Pharmaceutical Sciences Islamabad, Riphah International University, Islamabad 44000, Pakistan; 3 Swat College of Pharmaceutical Sciences, Swat 19200, Pakistan; fwd_shah@yahoo.com; 4 Department of Zoology, College of Science, King Saud University, Riyadh 11451, Saudi Arabia; helserehy@ksu.edu.sa; 5 Department of Health and Biological Sciences, Abasyn University Peshawar, Peshawar 25000, Pakistan; amin.ullah@abasyn.edu.pk; 6 Institute of Pathology, University Hospital of Cologne, 50923 Cologne, Germany; 7 Department of Bioinformatics, Hazara University, Mansehra 21120, Pakistan

**Keywords:** glioblastoma, gene expression genes, tumor necrosis factor, survival analysis, celecoxib, temozolomide

## Abstract

Background: Glioblastoma (GBM) harbors significant genetic heterogeneity, high infiltrative capacity, and patterns of relapse following many therapies. The expression of nuclear factor kappa-B (NF-κB p65 (RelA)) and signaling pathways is constitutively activated in GBM through inflammatory stimulation such as tumor necrosis factor-alpha (TNFα), cell invasion, motility, abnormal physiological stimuli, and inducible chemoresistance. However, the underlying anti-tumor and anti-proliferative mechanisms of NF-κB p65 (RelA) and TNFα are still poorly defined. This study aimed to investigate the expression profiling of NF-κB p65 (RelA) and TNFα as well as the effectiveness of celecoxib along with temozolomide (TMZ) in reducing the growth of the human GBM cell line SF-767. Methods: genome-wide expression profiling, enrichment analysis, immune infiltration, quantitative expression, and the Microculture Tetrazolium Test (MTT) proliferation assay were performed to appraise the effects of celecoxib and TMZ. Results: demonstrated the upregulation of NF-κB p65 (RelA) and TNFα and celecoxib reduced the viability of the human glioblastoma cell line SF-767, cell proliferation, and NF-κB p65 (RelA) and TNFα expression in a dose-dependent manner. Overall, these findings demonstrate for the first time how celecoxib therapy could mitigate the invasive characteristics of the human GBM cell line SF-767 by inhibiting the NF-κB mediated stimulation of the inflammatory cascade. Conclusion: based on current findings, we propose that celecoxib as a drug candidate in combination with temozolomide might dampen the transcriptional and enzymatic activities associated with the aggressiveness of GBM and reduce the expression of GBM-associated NF-κB p65 (RelA) and TNFα inflammatory genes expression.

## 1. Introduction

High-grade gliomas constitute the majority of malignant brain tumors and are known to develop from mutant glial or glial progenitor cells [1]. The most prevalent and deadly primary brain tumor, glioblastoma (GBM), accounts for 50% of all gliomas [2,3]. Because the overall survival, tumor cell invasion, and therapeutic response for GBM are so dismal, molecular variables likely play a crucial role in the available therapy options [4]. Genome-scale gene expression profiling enables the molecular analysis of intratumor variability, revealing molecular signatures reflecting underlying pathogenic mechanisms and molecular traits that may be related to survival [5]. The evolutionarily conserved transcription factors known as nuclear factor kappa B (NF-κB p65 (RelA)) proteins coordinate several important biological processes, including immunity, inflammation, cell death, and survival. An evolutionarily conserved Rel homology domain is shared by the five mammalian family members RelA (p65), RelB, c-Rel, NFKB1 (p105/p50), and NFKB2 (p100/p52), which promote DNA binding and dimerization with other NF-κB subunits [6,7]. It has been suggested that targeting NF-κB p65 (RelA) increases survival by promoting a tumor microenvironment (TME) that is less immunosuppressive and more receptive to immunomodulation. Tumor necrosis factor-alpha (TNFα) and the accompanying receptor superfamily have been linked to the development of GBM, according to a prior study [8]. The pro-inflammatory cytokine TNFα is linked to both pro- and anti-apoptotic responses through its signaling pathways [9]. Interestingly, constitutively produced TNFα promotes glioma cell invasion and motility by activating NF-κB p65 (RelA) [10]. Radiation therapy, chemotherapy, and surgical resection are the current therapeutic treatments that are most commonly used to treat GBM. However, the basic characteristic of GBM shows that cells typically invade the brain parenchyma. Additionally, one characteristic of GBM cells is their chemoresistance to TMZ. To combat the spread and invasion of tumor cells in GBM, more effective curative regimens are urgently needed. The prognosis for patients receiving the current standard of care is still quite dismal, with a five-year overall survival rate below 5% [11]. Unfortunately, clinical trials investigating immunotherapies have shown limited success in GBM patients [12]. Additionally, it has been shown that dysregulation of NF-κB signaling in human GBM enhances glioma cell survival, proliferation, and chemoresistance. [13]. In this regard, therapeutically disabling NF-κB p65 (RelA) expression and enzyme functioning seems like a better approach to disrupting the NF-κB p65 (RelA) inflammatory signaling cascade by preventing the spread and invasion of tumor cells. According to multiple investigations, celecoxib suppresses the development of tumor cells by interacting with several Cyclooxygenase-independent (COX) targets [14]. To treat recurrent malignant gliomas, celecoxib-based treatment therapies are in clinical trial phase I and phase II studies, and it has been determined that such combinations are safe [15,16]. Here, we hypothesized that celecoxib-mediated antineoplastic responses in GBM may prevent NF-κB p65 (RelA) activation due to its various roles in GBM. Celecoxib is a nonsteroidal anti-inflammatory drug (NSAID) and a selective inhibitor of cyclooxygenase-2 (COX-2). COX-2 is involved in the production of pro-inflammatory prostaglandins. Prostaglandins can activate the NF-κB pathway. Celecoxib inhibits COX-2 and prostaglandin synthesis. It could interfere with NF-κB activation downstream of COX-2 [13].

In this investigation, a comparative study of the mRNA expression of NF-κB p65 (RelA) and TNFα in both 33 brain tumor samples and TCGA datasets has revealed that the transcriptional activity of these genes is significantly higher in tumor samples than in normal samples. NF-κB p65 (RelA) and TNF-α were discovered to be significantly expressed in tumor samples of various cancers through pan-cancer expression analysis. This was followed by an investigation of these genes’ expression in the TCGA GBM datasets, and clinical biopsies of GBM patients confirmed the high expression of these respective genes. Additionally, functional enrichment analysis and immune infiltration were also carried out. After investigating the cytotoxic effects of TMZ and celecoxib in a GBM SF-767 cell line, the gene expression level of candidate genes was analyzed in a dose-dependent manner for both drugs to study their anti-inflammatory potential. Celecoxib’s impact on tumor cell invasion in glioblastoma by regulating NF-κB activation and mRNA expression has not been the subject of any studies to date. The aim of this study was to assess the celecoxib effect on invasive characteristics of the human glioblastoma cell line SF-767 by modifying the NF-κB cascade and NF-κB p65 (RelA) transcriptional levels. Our research findings provided evidence that the antineoplastic activity of celecoxib is mediated via NF-κB p65 (RelA) signaling suppression in glioblastoma. The current investigation can act as a springboard to examine the effects of radiation, TMZ, and celecoxib combination therapy in GBM patients.

## 2. Materials and Methods

### 2.1. Bioinformatics Analysis

The Cancer Genome Atlas (TCGA) and Genotype-Tissue Expression (GTEx) databases, a data collection and analysis repository on cancer [17], are accessible at https://portal.gdc.cancer.gov (accessed on 19 March 2022). The databases were used to ascertain the expression levels of NF-κB p65 (RelA) and TNFα in high-grade glioma (DNA and mRNA) by using the following criteria: *p*-value = 0.05, fold change 2, and top 10% gene rank for all data types. Using the TCGA and GTEx datasets and the integration of the c-Bioconductors R packages (Bioconductor version 3.17 software packages) with servers, i.e., the GEPIA platform GEPIA2 2019 Release notes (http://gepia.cancer-pku.cn/ (accessed on 19 March 2022)), gene expression profiles of several cancer types and pairs of normal samples were created [18]. By examining 9736 tumors and 8587 normal RNA sequencing samples, which were gathered from the TCGA and GTEx programs, the GEPIA web server GEPIA2 (2019 release note) (http://gepia.cancer-pku.cn (accessed on 19 March 2022)) was used to obtain the gene expression profile of NF-κB p65 (RelA) and TNFα among the majority of cancer types. Then, by contrasting it to 207 normal samples and 163 tumors from TCGA and GTEx, it was possible to assess the pattern of NF-B p65 (RelA) and TNFα expression in GBM. Furthermore, we examined the relationship between overall survival (OS) for GBM patients and the differential expression of NF-κB p65 (RelA) and TNFα in pan cancer analysis. *p* < 0.05 was regarded as statistically significant for the survival curve. The expression of NF-κB-p65 (RelA) and TNFα in GBM was utilized to create the overall survival (OS) curves; patients with a high level of expression (>median expression value) and patients with a low level of expression (<median expression value) were defined. The Kaplan–Meier (KM) method was used to evaluate overall survival using a log-rank test (statistically significant: *p*-value < 0.05) and to determine the hazards ratio (HR) with a 95% confidence interval [19]. In order to validate patient survival statistics, through UALCAN, we also ascertained the amounts of NF-κB p65 (RelA) and TNFα gene expression in GBM and also determined the functionality of genes that affected the patients’ survival times. We examined the NF-κB-p65 (RelA) and TNFα association profiles in healthy brain tissue and GBM samples using the UALCAN database (http://ualcan.path.uab.edu (accessed on 19 March 2022)). We discovered a connection between candidate gene expression levels and the grade of GBM tumors. Kaplan–Meier survival analysis was employed by UALCAN and provides survival curves, log-rank *p*-values, and HRs with 95% confidence intervals. The statistical significance for the survival curve was set at *p* < 0.05 [20]. TPM normalization for gene expression analysis was utilized by GEPIA and UALCAN [21,22] and adjusted by gene expression values by accounting for the total number of reads and the transcript length, allowing for accurate comparisons of gene expression levels across samples [23,24]. GeneMANIA was used in this study to analyze the networks and roles of the NF-κB p65 (RelA) and TNF α proteins. Through the GeneMANIA network, we accessed NF-κB p65 (RelA) and TNFα interactive genes [25]. Following that, functional studies of these genes were performed using FunRich and Metascape [26]. The expression levels of NF-κB p65 (RelA) and TNFα in GBM were measured using TIMER, and the relationship between these expression levels and immune infiltration levels in GBM was assessed [27].

### 2.2. Ethics Statement

The study was carried out in accordance with the Declaration of Helsinki, and it was approved by the Capital University of Science and Technology (CUST), Islamabad, Pakistan (Ref: BI and BS/ERC/19-2 and 23 September 2019). All the patients gave their verbal and written agreement to the use of their data for research purposes. The biopsy samples of 33 patients with glioblastoma (23 men, 10 women, median age 50 ± 13 years) who underwent brain surgery between January 2018 and December 2021 were obtained from various surgery departments of public sector tertiary care hospitals in Pakistan. None of the study subjects had received any radiotherapy or chemotherapy prior to sample collection.

#### Tissue Samples

The samples were initially obtained from patients primarily from the affected brain regions, specifically the frontal and temporal sites of the primary tumor, through surgical resections. The collection of tumor tissue samples was conducted by considering variations in cellularity and the presence of necrotic areas in patients with glioblastoma multiforme (GBM). Tumor-associated normal tissues (TANT) were typically obtained from the region adjacent to the tumor mass [28,29]. The minimum weight required for processing, as per internal guidelines, includes 125 mg of tumor tissue and 50 mg of adjacent normal tissue. Volumetric measurement was utilized to assess the size of GBM tumor samples. The tissue specimens were sectioned into small fragments (approximately 1–2 mm^3^ in size) through the utilization of a sterile scalpel. These fragments were subsequently subjected to preservation techniques involving formalin fixation and later paraffin embedding (FFPE) for the purpose of histopathological examination and immunohistochemistry. Additionally, the tissue fragments were appropriately stored at ultra-low temperatures (−80 °C) in order to maintain the integrity of nucleic acids and proteins and prevent degradation [30,31].

### 2.3. Quantitative qRT-PCR Analysis

In order to reduce degradation by ubiquitous DNases and RNases, bio specimens of glioblastoma designated for genomic analysis were microdissected and kept in the nucleic acid stabilizing reagent RNA later (Sigma-Aldrich, Cat No. R0901, Saint Louis, MI, USA). Specimens were immediately frozen in liquid nitrogen after ablation and stored at −80 °C until RNA extraction. Total RNA was extracted using the TriZol reagent (Thermo Fisher Scientific, Cat No, 15596018, Carlsbad, CA, USA). Superscript II reverse transcriptase (Invitrogen, Paisley, UK) was used to create cDNA with the cDNA synthesis kit (Thermo Fisher Scientific, Cat No. K1622, Vilnius, Lithuania), and the SYBR^®^ Green Master Mix kit (Maxima SYBR Green/ROX qPCR Master Mix (2×) (Thermo Fisher Scientific Cat No. K0221 Cat# K0221, Vilnius, Lithuania) was utilized for qPCR to amplify the particular products of PCR of all three genes presented in this work (Thermo Scientific, Carlsbad, CA, USA). Using a Nano Drop One spectrophotometer from Thermo Fisher Scientific, the purity of each RNA sample was determined. Reactions for each sample were performed in triplicate using a PCR protocol. Following 3 min of initial denaturation at 95 °C, the cycling conditions were 40 cycles consisting of denaturation at 95 °C for 10 s followed by annealing and extension at 60 °C for 30 s. The results were presented as CT values, defined as the threshold PCR cycle number at which an amplified product was first detected. The average CT value was calculated for both NF-κB p65 (RelA) and TNFα, and the ΔCT value was determined as the mean of the triplicate CT values. The 2^−ΔΔCT^ method was used to analyze the relative changes in gene expression [32,33]. The primers used for TNFα were (Forward Primer CCTCTCTCTAATCAGCCCTCTG and Reverse Primer GAGGACCTGGGAGTAGATGAG) and for NF-κB p65 (RelA) (Forward Primer AGGCAAGGAATAATGCTGTCCTG and Reverse Primer ATCATTCTCTAGTGTCTGGTTGG), and for β–actin (Forward Primer CATGTACGTTGCTATCCAGGC and Reverse Primer CTCCTTAATGTCACGCACGAT) [34,35].

### 2.4. ELISA

Prior to protein extraction, high-grade glioma biopsy samples were placed in sterile containers, frozen, and kept at −80 °C. The supernatants were slowly defrosted on ice. A total of 100 µL of supernatant was measured using 96-well enzyme-linked immunosorbent assays (ELISA). Protein-specific ELISA kits (Abcam Elisa kits USA) were used to measure the levels of the genes TNFα and NF-κB p65 (RelA) in accordance with the manufacturer’s instructions. The known concentrations of TNFα and NF-κB p65 (RelA) were added to the ELISA plate. The OD values obtained from the standards were used to plot a calibration curve, which interpolates protein concentrations based on their OD values. Blank correction was used to correct background noise, and OD values were obtained for each well of the ELISA plate at 450 nm as per the guidelines. Sample processing involved homogenization of glioblastoma tissue samples, extracting proteins of interest, incubation, washing, detection, and substrate addition. The specific details of the ELISA test procedure were followed by the specific kit and manufacturer’s instructions (Abcam Elisa Kits, Boston, MA, USA). Cytokine levels were assessed using the appropriate ELISA MAXTM Deluxe Set in accordance with the manufacturer’s guidelines (TNFα ELISA Kit, Cat. No. (ab181421), NF-κB p65 (RelA) ELISA Kit, Cat No. (ab176648)). The specific binding optical density at 450 nm was determined by a spectrophotometer [36].

#### Statistics

The results of the RT-PCR and ELISA data are expressed as the mean ± SD from at least three independent experiments for statistical analysis and were analyzed by GraphPad Prism 9 (Prism 9.5.0) software. The chi-square test and the two-tailed Student’s t-test were used to compare the two groups’ statistical significance. The D’Agostino and Pearson tests were used for the normality assessment.

**In vitro study of NF-κB p65 (RelA) and TNFα in the SF-767 human glioblastoma cell line.**

### 2.5. Cell Line and Culture Conditions

Human glioma cell line SF-767 was cultivated as monolayers in 75 cm^2^ tissue culture flasks in Iscove’s Modified Dulbecco’s Medium (IMDM), supplemented with 10% fetal bovine serum (FBS), 1% glutamine, 100 IU/mL penicillin, and 100 μg /mL streptomycin combination. Cell cultures were subcultured three times weekly and kept at 37 °C in a humidified 5% CO_2_ environment. Utilizing cell cultures at low passages, each assay using glioma cell lines was carried out separately in triplicate.

### 2.6. MTT Cellular Proliferation Assay

The antiproliferative impact of the therapy was assessed using the MTT assay (Roche Diagnostic GmbH, Basel, Switzerland). The yellow tetrazolium salt MTT [3-(4,5-dimethylthiazol-2-yl)-2,5-diphenyl tetrazolium bromide] can only be broken down into purple formazan crystals by metabolically active cells. Three repetitions of 10,000 cells/well in 200 μL medium were used to seed the 96-well culture plates. Approximately 10 μL of MTT reagents were added to each well following each treatment, and the plates were then incubated at 37 °C for 4 h. A spectrophotometer set at λ = 595 nm was used to measure the optical density (OD) after the cells had been lysed with 100 μL of solubilization buffer. Results are given as percentages compared to the control. The mean values acquired from the cell viability studies were statistically compared using the Student’s *t*-test in Microsoft Excel with one-tailed distributions. The analysis of variance (ANOVA) and *t*-test were used to examine the significance of differences between the study groups. Statistics were judged significant for values with *p* < 0.05. Results are presented as the mean standard deviation (SD) for all data. Each study was carried out in triplicate.

### 2.7. Quantitative Expression Analysis by qRT-PCR

To isolate total RNA, SF-767 cell line cells were treated with 50 µM, 100 µM, and 150 µM TMZ and celecoxib for 48 h in six-well plates. Trizol was used to extract the total RNA from SF-767 cell line cultures that had undergone control and stress. Using a nanodrop spectrophotometer, the RNA quantity was calculated at 260 nm absorbance. The RNA was cleaned in 1 mL of ethanol before being dissolved in 50 µM of water treated with 0.1% Diethyl Pyrocarbonate (DEPC) and stored at −80 °C until usage. According to the instructions provided by the manufacturer, 1 µL of RNA was reverse transcribed into cDNA using the RevertAidTM first-strand synthesis kit (Thermo Scientific, Cat No. K1622). As previously mentioned, the sequences of the forward and reverse primers (0.5 µM each) employed in the current study are listed below. The final reaction volume of 20 μL received the addition of the template cDNA (2 µL). qRT-PCR was carried out using a StepOne Plus thermocycler from Applied Biosystems and SYBER Green PCR Master Mix from Thermofisher (catalogue number K0221). Selected genes (NF-κB p65 (RelA) and TNFα) had their transcriptome expressions adjusted to the internal control GAPDH gene. All real-time PCR assays were carried out in triplicate, and the results were presented as the mean of three independent experiments to detect any significant differences between cells treated with TMZ, celecoxib, and untreated control cells. The results of each experiment, presented as the mean standard deviation, were carried out at least three times. Statistical evaluations were performed using GraphPad Prism 9 (Prism 9.5.0) software (GraphPad Software Inc., La Jolla, CA, USA). Data analysis employed one-way analysis of variance (ANOVA). The significance criterion for differences between means was set at *p* < 0.05.

## 3. Results

### 3.1. Expression Levels of NF-κB p65 (RelA) and TNFα in Different Cancer Types

**Figure 1:**
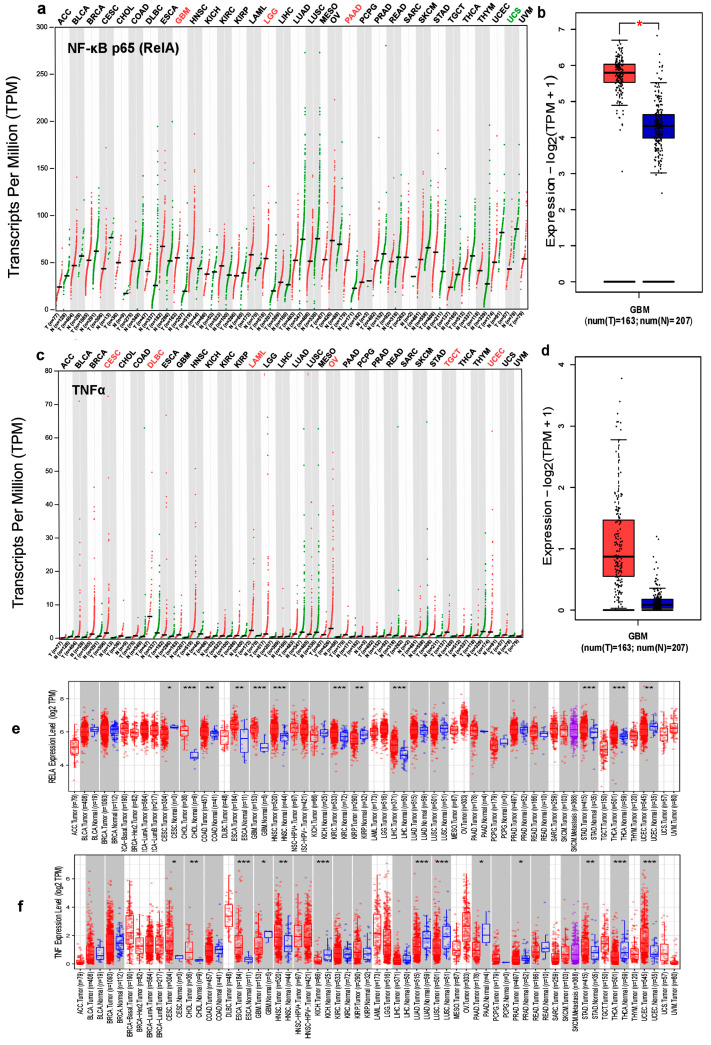
Expression levels of NF-κB p65 (RelA) and TNFα in various human cancers. (**a**) Expression profiles of NF-κB p65 (RelA) in tumors and paired normal tissue samples from the TCGA database in different cancer types. The NF-κB p65 (RelA) gene expression profiles across all tumor samples and paired normal tissues are shown in a dot plot, and each dot represents the expression profile in one sample. (**b**) Expression profiles of the NF-κB p65 (RelA) transcript (box plot); |log2FC| = 1 and * *p* < 0.01 in GBM and paired normal tissues from the GEPIA database. (**c**) The expression of TNFα in all tumor samples and paired normal tissues (dot plot). Each dot represents the expression of a sample. (**d**) Expression profiles of TNFα transcript (box plot); |log2FC| = 1 and * *p* < 0.01 in GBM and paired normal tissues from the GEPIA database. (**e**) Expression levels of the NF-κB p65 (RelA) gene in different cancer types compared to corresponding normal tissues from the TIMER database. Distributions of gene expression levels are displayed using box plots, with the statistical significance of differential expression evaluated using the Wilcoxon test. * *p* < 0.05, ** *p* < 0.01, and *** *p* < 0.001. (**f**) Expression levels of the TNFα gene in different cancer types compared to corresponding normal tissues from the TIMER database by using the Wilcoxon test. * *p* < 0.05, ** *p* < 0.01, and *** *p* < 0.001.

We found significant differences in the NF-κB p65 (RelA) and TNFα gene expression profiles between tumor and matched normal tissues using the TCGA database, which contained total unique analyses of about 163 tumor and 207 normal tissue samples. Contrary to normal tissues, higher levels of NF-κB p65 (RelA) expression were found in the following cancer types: CHOL, cholangiocarcinoma; ESCA, esophageal carcinoma; DLBC, lymphoid neoplasm diffuse large B-cell lymphoma; GBM, glioblastoma; HNSC, head and neck squamous cell carcinoma; KRIC, kidney renal clear cell carcinoma LGG, brain lower grade glioma; LIHC, liver hepatocellular carcinoma; MESO, mesothelioma; OV, ovarian serous cystadenocarcinoma; PAAD, pancreatic adenocarcinoma; TGCT, testicular germ cell tumors; SARC, sarcoma; STAD, stomach adenocarcinoma; THYM, thymoma; and THCA, thyroid carcinoma; conversely, lower levels of NF-κB p65 (RelA) expression were found in the following cancer types: ACC, adrenocortical carcinoma; BLCA, bladder urothelial carcinoma; CESC, cervical squamous cell carcinoma and endocervical adenocarcinoma; and COAD, colon adenocarcinoma (Figure 1a).

The expression of NF-κB p65 (RelA) in all tumor samples and paired normal tissues was also represented by a bar plot. The height of the bar represents the median expression of certain tumor types or normal tissue in ACC, adrenocortical carcinoma; BRCA, breast invasive carcinoma; CHOL, DLBC, GBM, KICH, kidney chromophobe; KIRP, kidney renal papillary cell carcinoma; LGG, LUAD, lung adenocarcinoma; OV, PCPG, pheochromocytoma and paraganglioma; READ, rectum adenocarcinoma; SKCM, skin cutaneous melanoma; TGCT, testicular germ cell tumor; and THYM, UCS, uterine carcinosarcoma (Supplementary Figure S1).

The NF-κB p65 (RelA) sequencing data by GEPIA also revealed increased expression in GBM transcripts per million, as demonstrated in Figure 1b. Similar to this, higher levels of TNFα expression were seen in the following cancer types: BLCA, BRCA, CESC, CHOL, COAD, DLBC, ESCA, GBM, HNSC, KRIP, KRIC, LAML, LGG, LIHC, MESO, OV, PAAD, PCPG, PRAD, READ, SARC, STAD, TGCT, UCEC, and UCS, while lower levels of TNFα expression were seen in the following cancer types (Figure 1c). Similarly, TNFα showed differential expression among different cancers through a bar plot (Supplementary Figure S2). Although TNFα expression was increased in a number of malignancies, it was shown that glioblastoma had the highest amount of enhanced TNFα expression. Then, using GEPIA, we examined the TNFα RNA sequencing data. The highest TNFα transcript expression levels per million were seen in GBM compared to matched normal tissues (Figure 1d). Additionally, GBM had the highest levels of NF-κB p65 (RelA) gene expression according to the TIMER database (Figure 1e). The GBM showed differential TNFα gene expression levels in the TIMER database (Figure 1f). These results demonstrated that the expression of NF-κB p65 (RelA) and TNFα in GBM was much higher than in normal tissues. As a result, it was worthwhile to investigate further the link between NF-κB p65 (RelA) and TNFα and related genes in the network of GBM since it may have a possible diagnostic value for GBM.

### 3.2. NF-κB p65 (RelA), TNFα and Survival in GBM

**Figure 2:**
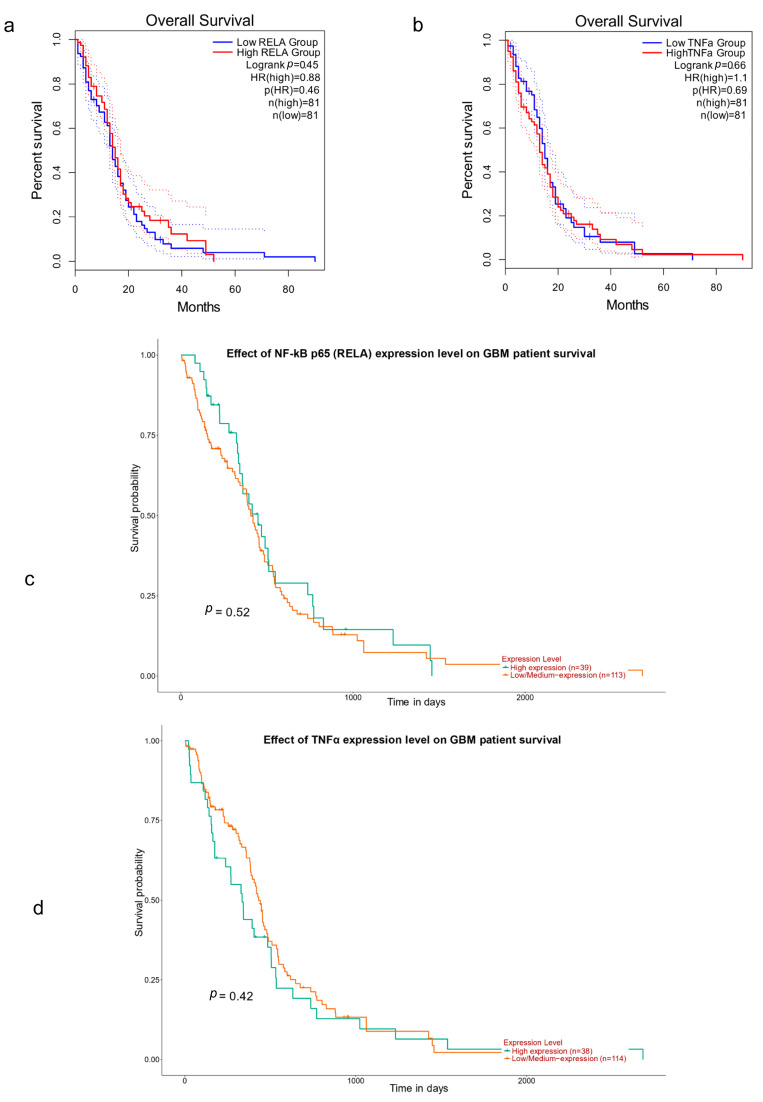
Comparisons of the effects of high and low expression levels of NF-κB p65 (RelA) and TNFα on the survival time of GBM patients using the GEPIA database. (**a**) Elevated expression levels of NF-κB p65 (RelA) were associated with poor OS; low NF-κB p65 (RelA) has a higher median survival in GBM patients. (**b**) Low expression of TNFα alpha has a higher median survival in GBM patients but is not statistically significant. The 95% CI is shown by the dotted lines in both (**a**,**b**). (**c**) Effect of NF-κB p65 (RelA) expression levels in UALCAN on the survival of GBM patients. (**d**) Effect of TNFα expression levels in UALCAN on the survival of GBM patients.

We assessed the predictive significance of NF-κB p65 (RelA) and TNFα in cancer using GEPIA to determine whether the expression levels of these proteins are connected to the prognosis of cancer patients. Although NF-κB p65 (RelA) and TNFα expression levels varied depending on the type of tumor, we found that the GBM exhibited an association between NF-κB p65 (RelA) and TNFα expression levels and overall survival time (OS). Furthermore, the result demonstrates that GBM with overexpression of NF-κB p65 (RelA) had a poor OS prognosis, and low levels of NF-κB p65 (RelA) had a higher median survival but were not statistically significant (Figure 2a). Similarly low levels of TNFα also showed higher median survival but were not statistically significant (Figure 2b). These genes were further investigated because of their trend towards poor survival with higher marker expression. Thus, we used global databases to compare and investigate the connection between these genes and GBM. NF-κB p65 (RelA) and TNFα overexpression were linked to poor survival outcomes in GBM patients, according to data from the UALCAN database, respectively. (Figure 2c,d), which, for the most part, coincided with the results from the GEPIA2 databases.

### 3.3. PPI Network and Functional Enrichment Analyses

**Figure 3:**
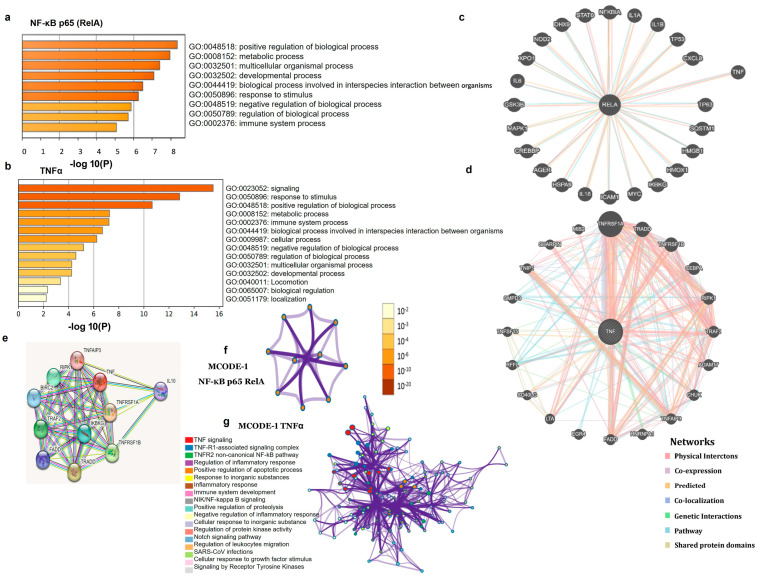
Proteinprotein interaction and functional enrichment analysis. (**a**) Clustered enrichment ontology categories (GO and KEGG terms) across the input gene NF-κB p65 (RelA)**,** colored by *p*-values by Metascape. (**b**) Metascape also constructed biological processes from the histogram of TNFα. (**c**–**e**) Gene interaction of NF-κB p65/RelA and TNFα by GeneMANIA and STRING. (**f**) The main biological processes in MCODE1 of NF-B p65 (RelA) involving the interacting genes are depicted using a cluster analysis from Metascape. In Metascape, MCODE complexes can be recognized automatically based on their IDs. Network representation of enriched biological pathways facilitates the connections between various biological processes. (**g**) Network of the enriched term of TNFα that was entered into this system for analysis. A circle node represents each term, and the node size is directly proportional to the number of input proteins grouped into each term. The node’s color denotes its cluster identity. GO terms with a similarity score >0.3 are connected by an edge, and the edge thickness represents the similarity score.

The NF-κB p65 (RelA) and TNFα proteins showed functional networks in PPI, which were primarily enriched in various functions. Metascape analyzed the biological functions of the NF-κB p65 (RelA) and TNFα interaction genes. We discovered that these genes strongly influenced response to stimuli, metabolism, biological regulation, the immune system, multicellular organismal processes, cellular component organization or biogenesis, and developmental processes (Figure 3a,b). The biological functions and gene interactions of NF-κB p65 (RelA) and TNFα were also assessed by GeneMANIA (Figure 3c,d), and the results were quite comparable to those of Metascape. This provided evidence of the molecular processes connected to the interaction between NF-κB p65 (RelA) and TNFα genes. The STRING database has been used to conduct protein-protein interaction enrichment analysis for each supplied gene list (Figure 3e). The subset of proteins that physically interact with at least one additional member of the list is found in the resulting network. The Molecular Complex Detection (MCODE) algorithm 10 has been used to discover densely connected network components if the network contains between 3 and 500 proteins. The MCODE networks for the specific genes MCODE 1 p-65 RELA and MCODE 1 TNF-α have been compiled and are displayed in (Figure 3f,g). Each MCODE component was subjected to pathway and process enrichment analysis separately, and the three terms with the highest *p*-values were kept as the functional descriptions of the associated components, as indicated in Table 1 underneath the relevant network plots in Figure 3f,g.

### 3.4. Correlation between Expression Levels and Immune Cell Infiltration Levels

**Figure 4:**
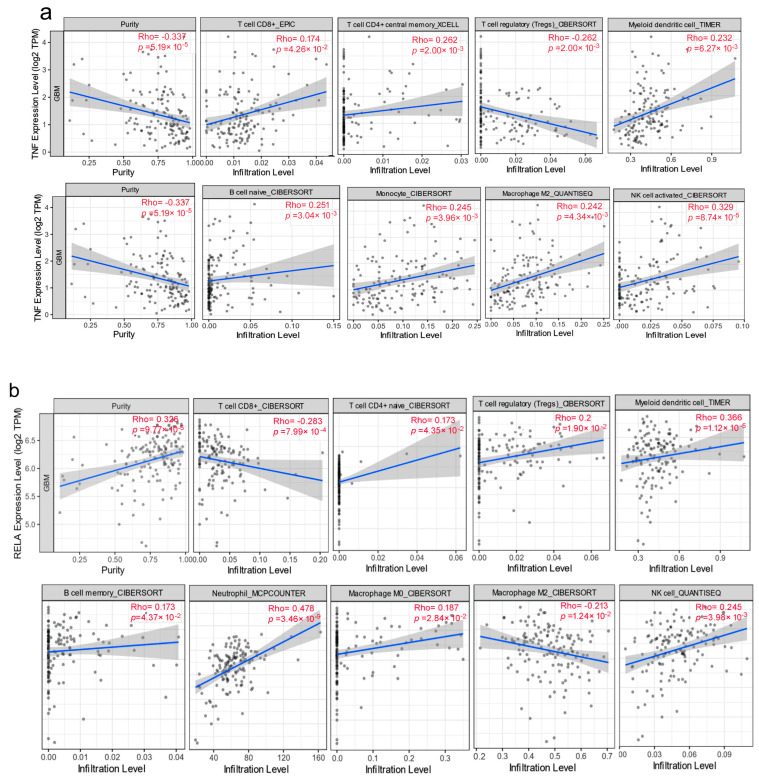
Relationships between NF-κB p65 (RelA) and TNFα expression levels and immune cell infiltration levels in GBM. Correlation between the abundance of immune cells and the expression of (**a**) TNFα and (**b**) NF-κB p65 (RelA).

We used the TIMER web server with the integration of EPIC, CELL, CIBERSORT, and QUANTISEQ to visualize the correlation between NF-κB p65 (RelA) and TNFα gene expression levels and immune infiltration levels in GBM. We found that the expression levels of TNFα were positively correlated with B cells, CD8 + T cells, CD4+, monocyte, macrophages, myeloid dendritic cells, and NK cell infiltration levels in GBM, and TNFα was also negatively correlated with Treg cells (Figure 4a). Similarly, NF-κB p65 (RelA) was positively correlated with CD4+, Treg cells, myeloid dendritic cells, B cells, Macrophage M0, NK cells, and neutrophils, and NF-κB p65 (RelA) was also negatively correlated with CD8 + T cells and Macrophage M2, as shown in Figure 4b.

### 3.5. Consistency of mRNA Expression Profiling and Validation in a GBM Patient Sample

**Figure 5:**
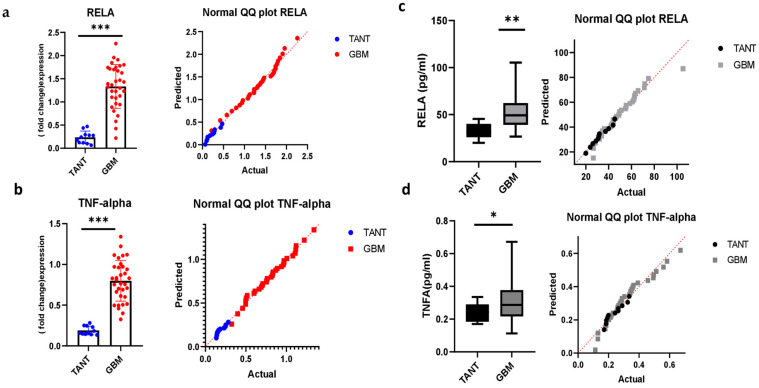
Quantification, expression, and verification of NF-κB p65 (RelA) and TNFα in glioblastoma patients. (**a**,**b**) Elevated expression levels of the two candidate genes in biopsy tissue of glioblastoma. Distributions of gene expression levels are displayed, with the statistical significance of differential expression evaluated using the *t*-test. *** *p* < 0.001. The values of three biological replicates are shown, indicating a univariate normality test. The graphs were plotted with the GraphPad Prism 9 (Prism 9.5.0) software. (**c**,**d**) An enzyme-linked immunosorbent assay validated the expression of DEGs in glioblastoma patients with univariate normalization using the *t*-test. ** *p* < 0.01 and * *p* < 0.05, respectively.

To evaluate the potential role of NF-κB p65 (RelA) and TNFα in glioblastoma, we quantified the expression of NF-κB p65 (RelA) and TNFα in 33 GBM samples. mRNA and protein expression of NF-κB p65 (RelA) and TNFα were significantly increased in glioblastoma biopsy samples. We examined the gene expression of targeted genes among glioblastoma specimen sections within the tumor and tumor-associated normal tissue (TANT). Tumor-associated normal tissue is obtained from the vicinity of the tumor site and serves as a comparison or control tissue for studying various aspects of tumor biology, including gene expression, signaling pathways, and cellular interactions. In the current study, it is obtained from the region adjacent to the tumor mass. All tissue samples were initially cut from four regions of the specimen, but only a selection with sufficient RNA quality and quantity was subjected to RT-PCR gene expression analysis. Normal QQ plots explain the same distribution and indicate the univariate normality of the dataset. NF-κB p65 (RelA) and TNFα exhibited increased expression in tumor tissue biopsy samples (Figure 5a,b). These data are also consistent with ELISA findings, with the indication of a univariate normality test. Each gene expression fold change FC was computed, and genes with |log2FC| > 2 and a *p*-value were identified (adjusted by the false discovery rate (Figure 5c,d)).

**In vitro expression study of NF-κB p65 (RelA) and TNFα in the SF-767 glioblastoma cell line after treatment with temozolomide and celecoxib.**

### 3.6. The Effect of Temozolomide and Celecoxib Treatment on Glioblastoma Cells

**Figure 6:**
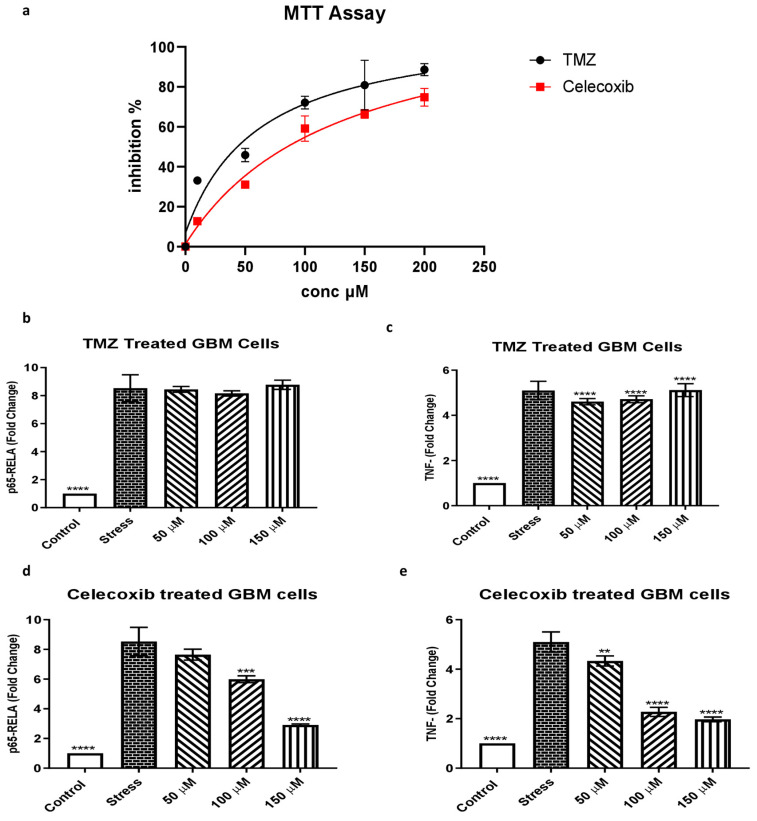
Assessment of cytotoxicity by MTT proliferation assay. The inhibition ratio of GBM cells after treatment with TMZ and celecoxib at different concentrations for 48 h. (**a**) Black and red curve TMZ and celecoxib in a dose-dependent manner. Increasing the concentration of any of these drugs makes the cytotoxic response more potent. The quantitative analysis of MTT is represented as the mean ± SD of three independent experiments. when compared to the untreated control. (**b**) Quantitative RT-PCR analysis of mRNA expression levels of the inflammatory marker NF-κB p65 (RelA) in the glioblastoma SF-767 cell line treated with TMZ at 50 µM, 100 µM, and 150 µM. The GAPDH gene was used as the internal control to normalize the data. IL-1B was used as stress to trigger an inflammatory cascade. The mRNA expression of genes was computed as a fold change compared to the control. The data are presented as the mean ± SD of the triplicate tests compared with the control group (*p* ≤ 0.05 for each). **** *p* < 0.0001. (**c**) Similarly, mRNA expression levels of the inflammatory marker TNFα in the glioblastoma SF-767 cell line treated with TMZ at 50 µM, 100 µM, and 150 µM. **** *p* < 0.0001. (**d**) Quantitative RT-PCR analysis of mRNA expression levels of NF-κB p65 (RelA) in the glioblastoma SF-767 cell line treated with celecoxib at 50 µM, 100 µM, and 150 µM. The GAPDH gene was used as the internal control to normalize the data. *** *p* < 0.001 and **** *p* < 0.0001. (**e**) Similarly, mRNA expression levels of TNFα in the glioblastoma SF-767 cell line treated with celecoxib at 50 µM, 100 µM, and 150 µM. ** *p* < 0.01 and **** *p* < 0.0001.

The TMZ treatment effects on the glioblastoma SF-767 cell line were evaluated for 48 h at concentrations of 10, 50, 100, 150, and 200 µM. The SF-767 cell line was exposed to TMZ 10 µM, and following the treatment, the cells were inhibited by 33.1%. A higher TMZ concentration (200 µM) was more lethal, resulting in 89% of the GBM cells dying following the treatment. Similarly, after being treated for 48 h with 10 μM of celecoxib, 12.8% of the cells died. In contrast, after being treated for 48 h with 200 µM of celecoxib, 75% of the cells died (Figure 6a). The results showed that cell viability decreased while increasing the concentration and duration of treatment. A higher dose of TMZ resulted in a higher cytotoxic effect in the MTT assay, as shown in Figure 6a.

Moreover, the expression of inflammatory biomarkers NF-κB p65 (RelA) and TNFα was studied after treatment with temozolomide and celecoxib. A quantitative RT-PCR analysis was performed in the SF-767 cell line treated with temozolomide at three concentrations (50 µM, 100 µM, and 150 µM) to assess the mRNA expression level of inflammatory genes (NF-κB p65 (RelA) and TNFα). Pro-inflammatory genes were significantly elevated in the stress groups after stimulation with IL-1 beta, a potent stimulator of inflammatory responses. The cells were treated for 48 hrs. Compared to the stress group, the TMZ did not significantly reduce the expression of NF-κB p65 (RelA) and TNFα in the glioblastoma SF-767 cell line in a dose-dependent manner (Figure 6b,c). Furthermore, the mRNA expression level of NF-κB p65 (RelA) and TNFα in the celecoxib-treated SF-767 cell line was studied at a similar concentration as TMZ. Compared to the stress group, celecoxib significantly reduced the expression of NF-κB p65 (RelA) and TNFα in the glioblastoma SF-767 cell line in a dose-dependent manner (Figure 6d,e).

## 4. Discussion

Glioblastoma (GBM) is an aggressive brain tumor with a more than 90% chance of recurrence. Determining biomarkers for GBM’s early diagnosis and prognosis is important. The NF-κB pathway transcription factor NF-κB p65 (RelA) and its related TNFα were found to be prospective targets in GBM by our comprehensive integrated approach through bioinformatics and clinical sample analysis. In this study, NF-κB p65 (RelA) and TNFα were found to be highly expressed in many tumor types, including GBM from global databases. In vitro glioblastoma’s ability to invade and infiltrate, NF-κB p65 (RelA) and TNFα play crucial roles, according to a wealth of evidence [37,38]. Therefore, we investigated a variety of datasets, including the Oncomine, GEPIA, and TIMER databases, to study the relationship between NF-κB p65 (RelA) and TNF-α expression in GBM. Our findings revealed that NF-κB p65 (RelA) expression levels were comparatively higher in GBM than in other tumors, while TNFα showed differential expression in GBM and other cancers through bioinformatics analysis. However, in the case of GBM, various proteins and signaling pathways are dysregulated, which could lead to NF-κB p65 (RelA) activation. TNFα is an extremely potent NF-κB p65 (RelA) activator. In the central nervous system (CNS), astrocytes, microglia, and certain neurons release the pro-inflammatory chemical TNFα. TNFα may indeed exhibit its effects through two receptors, TNFα receptors 1 and 2 (TNFR1 and TNFR2, respectively) [39]. The majority of cells typically express TNFR1, although oligodendrocytes and immune cells, particularly microglia, express TNFR2. Additionally, it was discovered that GBM and its associated endothelial cells expressed higher levels of TNFR1 compared to normal brain tissues and low-grade gliomas [40]. It is suggested that TNFα may be a possible diagnostic marker for GBM in response to that NF-κB signaling cascade. The dysregulation of numerous signaling pathways or growth factors and the triggering of a pro-inflammatory microenvironment in gliomas may lead to the activation of NF-κB p65 (RelA) [41,42]. High constitutive NF-κB p65 (RelA) activity is characteristic of GBM [43].

The impact of NF-κB p65 (RelA) and TNFα expression on the survival time of GBM patients was then assessed utilizing the UALCAN and GEPIA databases. These results revealed that high NF-κB p65 (RelA) and TNFα expressions were independent predictors of decreased OS for GBM. As in the previous studies, patients with GBM had shorter survival times due to upregulation of NF-κB p65 (RelA) and TNFα [44]. Nevertheless, constitutive NF-κB p65 (RelA) activation appears to promote the growth and metastasis of tumors by a range of mechanisms, including tumor metastasis, apoptosis, cell proliferation, angiogenesis, and metabolic reprogramming. It has been established that NF-κB p65 (RelA) stimulates the development of an inflammatory milieu that is conducive to the establishment of cancer [45]. Activation of constitutive NF-κB p65 (RelA) promotes survival and development in GBM.

These findings imply that these two genes can serve as a significant predictive marker for people with GBM [46]. Additionally, we used qRT-PCR to reanalyze the NF-κB p65 (RelA) and TNFα gene expression levels and transcripts by calculating the relative fold change in gene expression between a control sample (TANT) and experimental samples (samples from GBM patients) through the ΔΔCt normalizing method and discovered their higher levels. β-actin, also known as ACTB, is a commonly used reference gene for normalization in qRT-PCR. Due to its ubiquitous expression and consistent expression levels, it has been validated in previous studies that β-actin has been extensively used as a reference gene in numerous studies, including glioblastoma research. Its selection as a reference gene for normalization in glioblastoma qRT-PCR experiments is based on its consistent and reliable expression across samples [47]. It was inferred that NF-κB p65 (RelA) and TNFα might be an early diagnostic marker for GBM patients since the expression trend of the NF-κB p65 (RelA) and TNFα proteins was essentially compatible with the transcript [48]. The PPI network of NF-κB p65 (RelA) and TNFα by GeneMANIA has been investigated, and the biological processes associated with NF-κB p65 (RelA) and TNFα interaction genes were analyzed with Metascape in order to better understand why elevated expression levels of NF-κB p65 (RelA) and TNFα are significant for the poor prognosis of GBM patients. However, it is justified by different previous studies that NF-κB p65 (RelA) and TNFα are biologically plausible candidates and play an important role in inflammatory processes involved in various cancers, including GBM [49]. A poor prognosis can be associated with elevated levels of markers including C-reactive protein (CRP) and interleukin-6 (IL-6), which indicate systemic inflammation and link it to tumor aggressiveness and decreased overall survival [50]. Secondly, the release of pro-inflammatory cytokines by immune cells involved in inflammation that are linked to tumors, such as macrophages and microglia, contributes to the development of GBM and the disease’s resistance to treatment. Inflammatory mechanisms like the NF-κB pathway are activated during GBM growth and therapy. As a result, both systemic and tumor-specific inflammation are associated with a poor prognosis for GBM patients [51,52].

In the current study, we hypothesized that the biological functions of NF-κB p65 (RelA) and TNFα were connected with immunological processes, resulting in poor prognosis with elevated NF-κB p65 (RelA) and TNFα expression levels in GBM. Based on this presumption, TIMER was employed to examine the correlation between NF-κB p65 (RelA) and TNFα expression levels and immune cell infiltration levels in GBM. Numerous studies highlight NF-κB p65 (RelA) and TNFα mediated exacerbation of inflammation in the tumor microenvironment [53,54].

The NF-κB p65 (RelA) family of pleiotropic transcription factors is sequestered in the cytoplasm of most normal cells by noncovalent interaction. Recent investigations have demonstrated that various tumor cells express NF-κB p65 (RelA) constitutively activated [55]. Interestingly, in glioblastoma, TNFα induces tumor cell motility and invasion via activating NF-κB [56]. As anticipated, TNFα increased SF-767 cell invasion because of other metabolic stimuli due to the presence of LDL protein and receptors, which increased the cell proliferation turnover of growing tumor cells in this study and caused NF-κB p65 (RelA) activation. SF-767 cells revealed high-affinity LDL binding and maximum binding capacity [57]. Our results were categorically established in GBM SF-767 cells with NF-κB p65 (RelA) overexpression and silencing as a positive modulator of NF-κB signaling by enhancing the translation of the p65 transcript. Temozolomide (TMZ), an oral alkylating cytostatic medication, is frequently used to treat GBM; however, over 50 percent of individuals who use it do not experience any benefits [58].

Therefore, the SF-767 glioblastoma cell line was used for in vitro analysis in the current study. According to the data, when the SF-767 cell line was exposed to 10 µM TMZ, the inhibition was 33.1%. A higher concentration of TMZ (200 M) proved to be lethal in GBM cells, resulting in 89% cell death after the treatment. Due to the heterogeneity of the GBM tumor and its highly angiogenic and metastatic characteristics, combination therapies are now regarded as an essential component of anticancer therapy. Cancer monotherapy has become a rare chemotherapeutic treatment choice [59]. The standard treatment for GBM is temozolomide therapy combined with surgery and radiation therapy, but because this approach has minimal effect on patients’ overall survival, it is crucial to create drugs that can maximize their advantages and prevent tumor resistance [60]. The combination of TMZ with celecoxib would be a workable strategy to treat GBM, even though TMZ has been successful in treating GBM. Celecoxib can inhibit NF-κB p65 activation, combat the pro-inflammatory milieu, and improve therapeutic outcomes. The synergistic effects of celecoxib and TMZ on GBM cells’ apoptosis indicate that celecoxib may improve the effectiveness of TMZ, the current standard treatment for GBM [61]. Combining celecoxib with conventional therapies such as radiation or chemotherapy may be able to potentially target cancer stem cells [62]. Celecoxib additionally enhanced the effects of glucocorticoids triggering apoptosis in GBM cells by suppressing cyclooxygenase-2 (COX-2), which resulted in Akt-mediated activation of NF-κB and subsequent apoptosis [63,64]. Previous studies provided evidence that combining celecoxib with existing treatment modalities, such as TMZ or glucocorticoids, can have synergistic effects in GBM cells. These findings support the potential benefits of celecoxib as an adjunctive therapy to enhance the effectiveness of GBM treatments currently being used. However, mounting evidence pointing to NSAIDs’ wide variety of COX-dependent targets, such as the presence of NF-κB, B-CATENIN, PPAR DELTA, NAG-1, and BCL-2, suggests that various molecular pathways are implicated in the anticancer effect of these medications [65]. Understanding the regulation of NF-κB in cancer has led to the exploration of novel therapeutic approaches. Studies have demonstrated the inhibitory effects of celecoxib on the activation of the NF-κB signaling pathway, which is implicated in both inflammatory responses and the progression of tumors [13]. Celecoxib has exhibited potential anti-cancer effects in both preclinical and clinical studies by virtue of its capacity to modulate NF-κB activity. Furthermore, celecoxib has received approval for therapeutic use in colon carcinogenesis, rheumatoid arthritis, and various inflammatory disorders. Studies have demonstrated its ability to induce apoptosis and inhibit angiogenesis [66]. Celecoxib has been investigated as an adjunct therapy for certain cancer types, including colorectal cancer and pancreatic cancer. Prior studies have demonstrated that the utilization of celecoxib, alongside conventional chemotherapy protocols, has the potential to augment treatment efficacy and enhance overall patient survival rates [67,68]. The potential of celecoxib to impact crucial cellular processes associated with tumor growth, angiogenesis, and metastasis lies in its ability to target NF-κB.

Moreover, the investigation of NF-κB regulation and its modulation in specific cancer types has provided insights into potential therapeutic strategies beyond traditional chemotherapy. Targeted therapies aimed at inhibiting NF-κB signaling have been explored as a means to overcome drug resistance and improve treatment outcomes in cancers such as lymphoma, multiple myeloma, and breast cancer [69,70]. These studies highlight the potential clinical significance of targeting NF-κB in specific cancer contexts. Further research is needed to establish celecoxib’s clinical efficacy, determine optimal treatment strategies, and identify biomarkers for personalized patient selection. In this study, we offer evidence that celecoxib inhibits NF-κB activation while inhibiting the development of GBM cells. The effectiveness of TMZ and COX-2 inhibitors in treating GBM in vivo and in vitro has been demonstrated in earlier research, but the underlying molecular mechanism has not been clarified [71]. However, it has recently been found that the NSAIDs indomethacin and flurbiprofen suppress the growth of glioma cells [72,73]. Celecoxib, a medication used to treat inflammation, is now also used to treat cancer. There is growing evidence that, despite being a selective inhibitor of COX-2, it exerts anti-tumor effects on cancer cells that do not contain the COX-2 enzyme. In order to determine if celecoxib alone or in conjunction with other drugs is beneficial in treating glioblastoma, several researchers are now engaged in Phase II clinical studies [13]. Celecoxib and temozolomide were also used to treat a rat orthotropic glioma model, proving that both medications work well together to treat gliomas [15]. Our research supports the in vitro findings, but mounting evidence points to NSAIDs’ wide spectrum of COX-independent targets, such as NF-κB p65 (RelA) and TNFα, indicating that a number of molecular pathways may be involved in the inhibit-neoplastic action of these drugs. In the present research, we provide evidence that celecoxib suppresses the growth of GBM cells by inhibiting NF-κB activation and its signaling pathway. Additionally, individuals with glioblastoma receiving temozolomide, dexamethasone, and cranial radiation therapy for peritumoral brain edema could take celecoxib without any danger [74]. Celecoxib use has increased due to these trials, offering a desirable anti-glioma treatment plan. However, previous studies also highlighted some of the side effects and limitations of celecoxib use. Including cardiovascular risks, gastrointestinal effects, renal complications, allergic reactions, and drug interactions [75,76]. Additionally, a recent study revealed some inconsistencies regarding COX-2 inhibitors and GBM invasion, and contraindications regarding COX-2 inhibitors and GBM invasion have been reported [73]. To manage potential adverse effects and ensure patient wellbeing, adequate monitoring, an appropriate dose, and regular follow-up are important [77,78]. The study’s findings could be strengthened by considering the potential influence of age, gender, ethnicity, and environmental factors on the effects of COX-2 inhibitors in glioblastoma. These factors may contribute to variations in treatment response and outcomes, and exploring their impact could provide valuable insights for personalized medicine approaches.

In summary, our findings showed that celecoxib exhibits inhibitory effects on NF-κB activation, which is associated with inflammation, and it also hampers proliferation and triggers apoptosis in GBM cells. These findings highlight the potential of celecoxib as a therapeutic agent in the treatment of GBM.

## 5. Conclusions

The expression profile of NF-κB p65 (RelA) and TNFα in GBM patients was studied using the TCGA database and biopsy samples from glioblastoma patients who underwent surgery. We found high expression of these genes in GBM patients. NF-κB is a ubiquitous transcription factor that regulates the response to a diverse range of stimuli. Our research contributes to the individualized prognostic management of glioblastoma patients and provides evidence for targeting NF-κB and TNF family members. With over 90 percent of recurrent glioblastoma responding poorly to a second line of chemotherapy, acquired resistance to chemotherapy is a severe consequence of temozolomide therapy. This study explored the inhibitory effect of celecoxib on decreasing the expression of NF-κB p65 (RelA) and TNFα in the GBM cell line in comparison with TMZ. Our findings imply that celecoxib reduces the expression of Nfkb linked to suppression of COX2, hence reducing the proliferation of glioblastoma. Temozolomide therapy has a greater effect on cell viability. In the future, if these drugs are used in combination, they may show a synergistic effect by decreasing cellular proliferation and cell viability by celecoxib and temozolomide, respectively, against the SF-767.

## Figures and Tables

**Table 1:** MCODE components of NF-κB p65 (RelA) and TNFα by *p*-value as the functional description of the corresponding component network plots.

MCODE	GO	Description	Log10(P)
MCODE_1 p-65 RELA	GO:1902895	Positive regulation of miRNA transcription.	−8.3
MCODE_1 p-65 RELA	GO:2000630	Positive regulation of miRNA metabolic process.	−8.1
MCODE_1 p-65 RELA	WP4329	miRNAs involvement in the immune response in sepsis.	−8
MCODE_1 TNFα	M128	PID TNF Pathway.	−21.9
MCODE_1 TNFα	R-HSA-5357905	Regulation of TNFR1 signaling.	−21.7
MCODE_1 TNFα	R-HSA-75893	TNF signaling.	−21.1

## Data Availability

The data presented in this study are available on request from the corresponding authors.

## Supplementary Materials

The following supporting information can be downloaded at: https://www.mdpi.com/article/10.3390/jcm12206683/s1, Figure S1: The expression of NF-κB p65 in all tumor samples and paired normal tissues (bar plot). The height of bar represents the median expression of certain tumor type or normal tissue in ACC, BRCA, CHOL, DLBC, GBM, KICH, KIRP, LGG, LUAD, OV, PCPG, READ, SKCM, TGCT, THYM and UCS; Figure S2: The expression of TNFα in all tumor samples and paired normal tissues (bar plot). The height of bar represents the median expression of certain tumor type or normal tissue same as all cancer mentioned in NF-κB p65 (RelA).
