# Supplementary material for: Celecoxib Suppresses NF-κB p65 (RelA) and TNFα Expression Signaling in Glioblastoma
Source: J Clin Med. 2023 Oct 23;12(20):6683. doi: 10.3390/jcm12206683 (PMC10607796; doi:10.3390/jcm12206683)
Supplement: Supplementary file 1 [file jcm-12-06683-s001.zip › jcm-2457631-supplementary.pdf]

Supplementary Figures

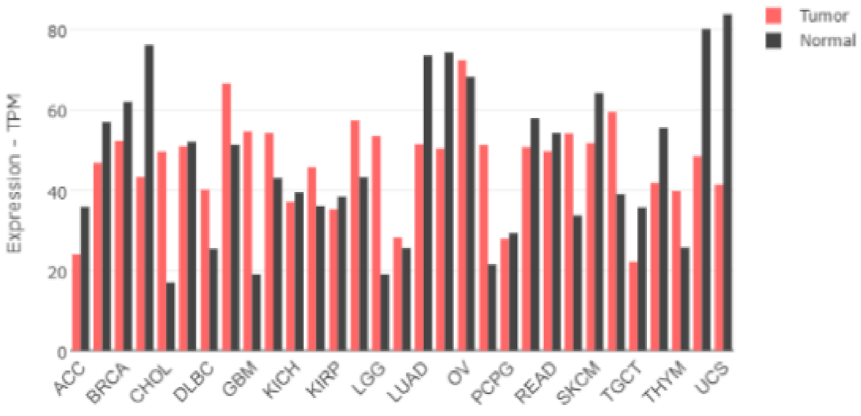

Figure S1: The expression of NF-κB p65 in all tumor samples and paired normal tissues (bar plot). The height of bar represents the median expression of certain tumor type or normal tissue in ACC, BRCA, CHOL, DLBC, GBM, KICH, KIRP, LGG, LUAD, OV, PCPG, READ, SKCM, TGCT, THYM and UCS.

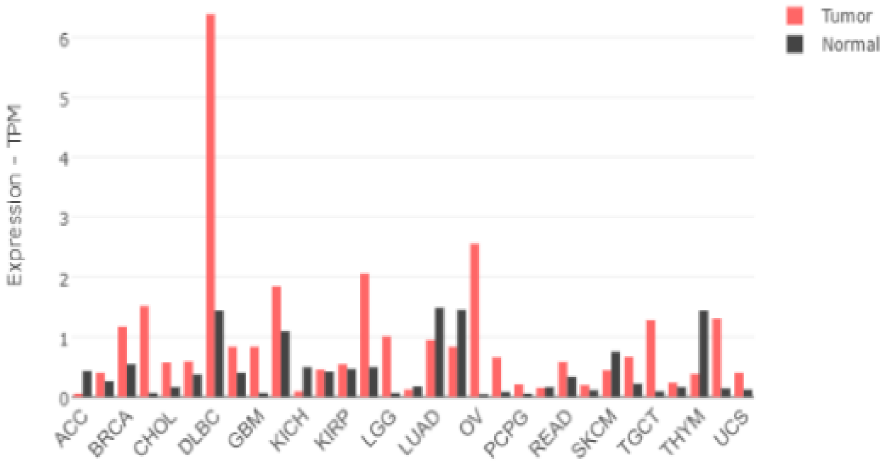

Figure S2: The expression of TNFα in all tumor samples and paired normal tissues (bar plot). The height of bar represents the median expression of certain tumor type or normal tissue same as all cancer mentioned in NF-κB p65 (RelA).
